# Supplementary material for: OsASR6 Enhances Salt Stress Tolerance in Rice
Source: Int J Mol Sci. 2022 Aug 19;23(16):9340. doi: 10.3390/ijms23169340 (PMC9408961; doi:10.3390/ijms23169340)
Supplement: Supplementary file 1 [file ijms-23-09340-s001.zip › ijms-1836560-supplementary.pdf]

Supplementary Materials

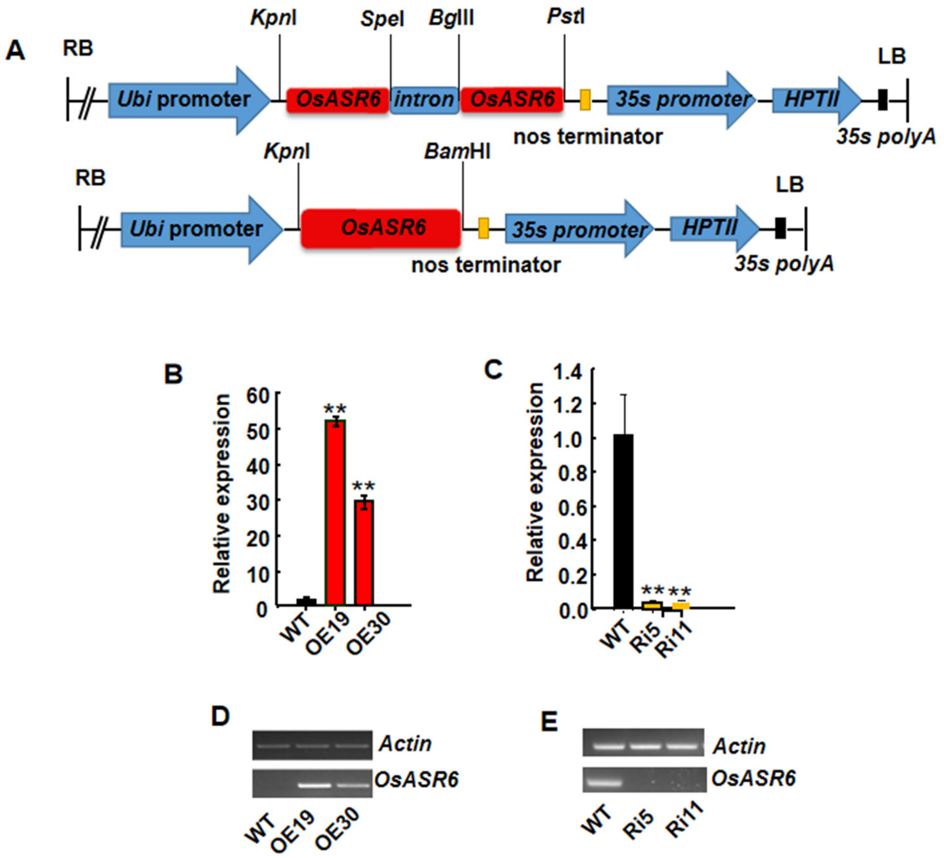

Figure S1. Plasmid construction and expression levels of *OsASR6* in transgenic rice. (**A**) Schematic representation of the RNAi (up) and overexpression (down) constructs used for the transformation of *OsASR6* transgenic rice. (**B**) Expression levels of *OsASR6* in WT and OE plants as determined by qRT-PCR. (**C**) Expression levels of *OsASR6* in WT and RNAi plants as determined by qRT-PCR. (**D**) Expression levels of *OsASR6* in WT and OE plants as determined by RT-PCR. (**E**) Expression levels of *OsASR6* in WT and RNAi plants as determined by RT-PCR. Rice *Actin1* was used as an internal control. Asterisks indicate significant differences between means of WT and transgenic lines. Data represent means ± SD. ** $p < 0.01$.

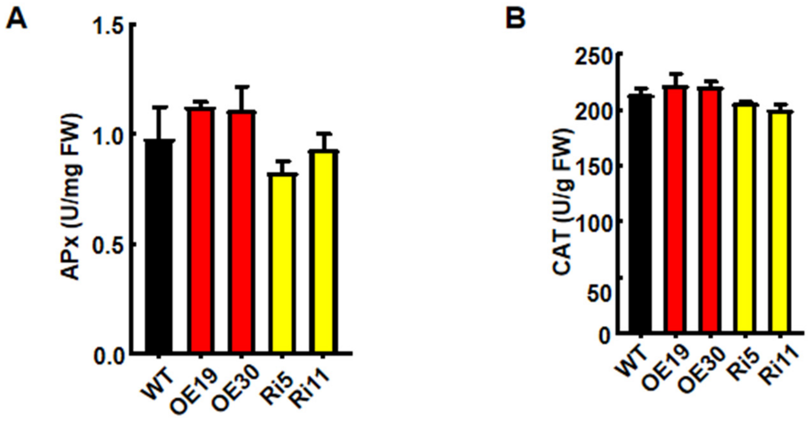

Figure S2. Analysis of APx and CAT activity in WT and *OsASR6* transgenic plants under salt treatment. (**A**) Apx enzyme activity, (**B**) CAT enzyme activity.
